# Workingmen and Drink

**Published:** 1889-08

**Authors:** 


### WORKINGMEN AND DRINK.

In the city of New York alone it is estimated that not less than $250,000 a day are spent for drink ; $1,500,000 in one week ; $75,000,000 in one year. Who will dispute it when I say that one-half of the policemen of New York City are employed to watch the beings who squander $75,000,000 a year ? Who will dispute it when I say that the money spent in paying the salaries and expenses of one-half of the police of New York could be saved to the taxpayers if $75,000,000 were not devoted to making drunkards, thieves, prostitutes, and other subjects for the policemen's nets to gather in ? If $250,000 go over the counters of the rumseller in one day in New York City alone, who will dare to assert that workingmen do not pay one fifth, or $50,000 of that sum ? If workingmen in New York City spend $50,000 a day for drink, they spend $300,000 a week, leaving Sunday out. In four weeks they spend $1,200,000—over twice as much money as was paid into the general assembly of the Knights of Labor in nine years. In six weeks they spend $1,800,000, nearly three times as much money as that army of organized workers, the Knights of Labor, have spent from the day the general assembly was first called to order up to the present day ; and in one year the workingmen of New York City alone will have spent for beer and rum $15,600,000, or enough to purchase and equip a first-class telegraph line of their own ; $15,600,000—enough to invest in such co-operative enterprises as would forever end the strike and lock-out as a means of settling disputes in labor circles.—*Medical Summary.*

